# 1-(Piperidin-1-yl)-3-(2,4,6-trimethyl­phen­yl)propan-2-ol

**DOI:** 10.1107/S1600536811006568

**Published:** 2011-02-26

**Authors:** Abel M. Maharramov, Ali N. Khalilov, Atash V. Gurbanov, Mirze A. Allahverdiyev, Seik Weng Ng

**Affiliations:** a Department of Organic Chemistry, Baku State University, Baku, Azerbaijan; b Department of Chemistry, University of Malaya, 50603 Kuala Lumpur, Malaysia

## Abstract

The title compound, C_17_H_27_NO, features a bufferfly-shaped substituted 2-propanol having an aromatic ring on the 1-carbon and a piperidine ring on the 3-carbon. The piperidine ring adopts a chair conformation and its N atom shows a trigonal coordination. In the crystal, the hy­droxy group inter­acts with the N atom of an inversion-related mol­ecule, generating an O—H⋯N hydrogen-bonded dimer.

## Related literature

For background to the synthesis: see: Yadigarov *et al.* (2010 ▶). For the structure of tolperisone hydro­chloride, see: Tanaka & Hirayama (2007 ▶). For a related structure, see: Maharramov *et al.* (2011 ▶). 
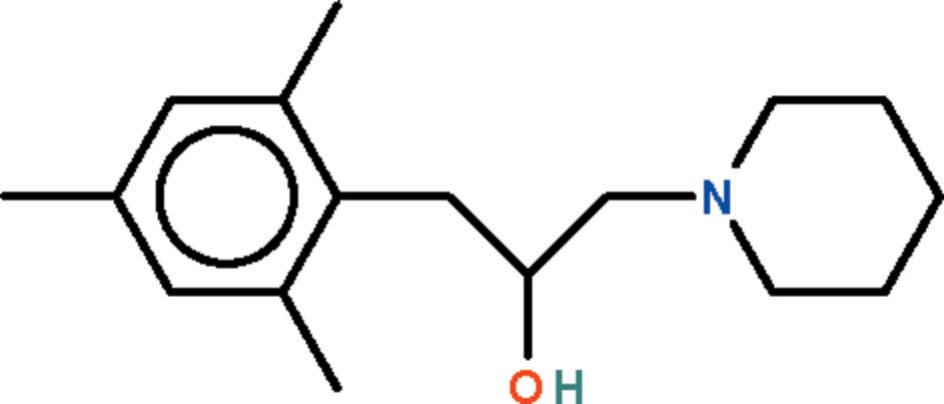


## Experimental

### 

#### Crystal data

C_17_H_27_NO
*M* *_r_* = 261.40
Monoclinic, 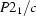

*a* = 11.7992 (12) Å
*b* = 8.0940 (8) Å
*c* = 17.0196 (17) Å
β = 107.489 (1)°
*V* = 1550.3 (3) Å^3^
*Z* = 4
Mo *K*α radiation
μ = 0.07 mm^−1^
*T* = 100 K
0.30 × 0.20 × 0.20 mm

#### Data collection

Bruker APEXII diffractometer
6615 measured reflections
3537 independent reflections
2964 reflections with *I* > 2σ(*I*)
*R*_int_ = 0.016

#### Refinement

*R*[*F* ^2^ > 2σ(*F* ^2^)] = 0.040
*wR*(*F* ^2^) = 0.110
*S* = 1.02
3537 reflections
179 parameters
1 restraint
H atoms treated by a mixture of independent and constrained refinement
Δρ_max_ = 0.36 e Å^−3^
Δρ_min_ = −0.19 e Å^−3^

### 

Data collection: *APEX2* (Bruker, 2005 ▶); cell refinement: *SAINT* (Bruker, 2005 ▶); data reduction: *SAINT*; program(s) used to solve structure: *SHELXS97* (Sheldrick, 2008 ▶); program(s) used to refine structure: *SHELXL97* (Sheldrick, 2008 ▶); molecular graphics: *X-SEED* (Barbour, 2001 ▶); software used to prepare material for publication: *publCIF* (Westrip, 2010 ▶).

## Supplementary Material

Crystal structure: contains datablocks global, I. DOI: 10.1107/S1600536811006568/bt5478sup1.cif

Structure factors: contains datablocks I. DOI: 10.1107/S1600536811006568/bt5478Isup2.hkl

Additional supplementary materials:  crystallographic information; 3D view; checkCIF report

## Figures and Tables

**Table 1:** Hydrogen-bond geometry (Å, °)

*D*—H⋯*A*	*D*—H	H⋯*A*	*D*⋯*A*	*D*—H⋯*A*
O1—H1⋯N1^i^	0.85 (1)	2.07 (1)	2.880 (1)	158 (2)

Symmetry code: (i) 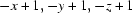.

**Fig. 1.**
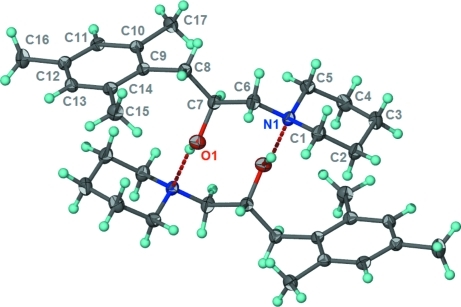
Anisotropic displacement ellipsoid plot (Barbour, 2001) of the hydrogen-bonded dimeric structure of C17H27NO at the 70% probability level; hydrogen atoms are drawn as spheres of arbitrary radius.

## supplementary crystallographic information

### Comment

A recent study reported the reaction of 1-chloro-3-(2,4,6-trimethylphenyl)propan-2-one and primary amines. The chlorine atom in the α-chloro ketone is not replaced directly by the amino RNH– group; the intermediate product undergoes a Favorskii rearrangement that furnishes a compound having two methylene groups between the aromatic system and the amido unit (Yadigarov *et al.*, 2010). A recent study used thiourea as the amino reactant (Maharramov *et al.*, 2011). The present study employs a cyclic secondary amine as the amino reactant in the synthesis of a compound having a formulation similar to that of tolperisone (a piperidine derivative that is commercially used as a muscle relaxant), which has been characterized as a hydrochloride (Tanaka & Hirayama, 2007). The title compound, C_17_H_27_NO,  (Scheme I) is a bufferfly-shaped substituted 2-propanol having an aromatic ring on one carbon end and a piperidinyl ring on the other. The hydroxy group interacts with the N atom of an inversion-related molecule to generate a hydrogen-bonded dimer (Fig. 1).

### Experimental

1-Chloro-3-(2,4,6-trimethylphenyl)propan-2-one (1 mol) and piperidine (1 mmol) were stirred in water for 18 h at 53 K. The water was decanted and the oil was distilled in vacuum. The distallate was a liquid; the liquid crystallized after 6 months; yield 70%.

### Refinement

Carbon-bound H-atoms were placed in calculated positions [C–H 0.95 to 0.99 Å; *U*(H) 1.2 to 1.5*U*(C)] and were included in the refinement in the riding model approximation, with *U*(H) set to 1.2 to 1.5*U*(C).

The hydroxy H-atom was located in a difference Fourier map, and was refined with a distance restraint of O–H 0.84±0.01 Å.

### Crystal data

**Table:**

C_17_H_27_NO	*F*(000) = 576
*M**_r_* = 261.40	*D*_x_ = 1.120 Mg m^−^^3^
Monoclinic, *P*2_1_/*c*	Mo *K*α radiation, λ = 0.71073 Å
Hall symbol: -P 2ybc	Cell parameters from 1896 reflections
*a* = 11.7992 (12) Å	θ = 2.8–29.2°
*b* = 8.0940 (8) Å	µ = 0.07 mm^−^^1^
*c* = 17.0196 (17) Å	*T* = 100 K
β = 107.489 (1)°	Prism, colorless
*V* = 1550.3 (3) Å^3^	0.30 × 0.20 × 0.20 mm
*Z* = 4	

### Data collection

**Table:**

Bruker APEXII diffractometer	2964 reflections with *I* > 2σ(*I*)
Radiation source: fine-focus sealed tube	*R*_int_ = 0.016
graphite	θ_max_ = 27.5°, θ_min_ = 2.5°
φ and ω scans	*h* = −13→15
6615 measured reflections	*k* = −8→10
3537 independent reflections	*l* = −15→22

### Refinement

**Table:**

Refinement on *F*^2^	Primary atom site location: structure-invariant direct methods
Least-squares matrix: full	Secondary atom site location: difference Fourier map
*R*[*F*^2^ > 2σ(*F*^2^)] = 0.040	Hydrogen site location: inferred from neighbouring sites
*wR*(*F*^2^) = 0.110	H atoms treated by a mixture of independent and constrained refinement
*S* = 1.02	*w* = 1/[σ^2^(*F*_o_^2^) + (0.0565*P*)^2^ + 0.4799*P*] where *P* = (*F*_o_^2^ + 2*F*_c_^2^)/3
3537 reflections	(Δ/σ)_max_ = 0.001
179 parameters	Δρ_max_ = 0.36 e Å^−^^3^
1 restraint	Δρ_min_ = −0.19 e Å^−^^3^

### Fractional atomic coordinates and isotropic or equivalent isotropic displacement parameters (Å^2^)

**Table:**

	*x*	*y*	*z*	*U*_iso_*/*U*_eq_	
O1	0.42350 (7)	0.36548 (10)	0.53159 (5)	0.0213 (2)	
H1	0.4165 (15)	0.373 (2)	0.4804 (6)	0.042 (5)*	
N1	0.54620 (8)	0.67399 (12)	0.62908 (6)	0.0164 (2)	
C1	0.64459 (10)	0.66941 (15)	0.70653 (7)	0.0196 (2)	
H1A	0.6716	0.5539	0.7190	0.023*	
H1B	0.6161	0.7100	0.7522	0.023*	
C2	0.74826 (10)	0.77528 (15)	0.70081 (7)	0.0219 (3)	
H2A	0.7807	0.7296	0.6580	0.026*	
H2B	0.8121	0.7723	0.7541	0.026*	
C3	0.70960 (11)	0.95379 (15)	0.67944 (7)	0.0229 (3)	
H3A	0.7759	1.0172	0.6697	0.027*	
H3B	0.6890	1.0054	0.7261	0.027*	
C4	0.60210 (11)	0.95923 (15)	0.60243 (7)	0.0225 (3)	
H4A	0.5717	1.0738	0.5930	0.027*	
H4B	0.6263	0.9249	0.5540	0.027*	
C5	0.50422 (10)	0.84561 (14)	0.61153 (7)	0.0196 (2)	
H5A	0.4751	0.8863	0.6568	0.024*	
H5B	0.4369	0.8481	0.5601	0.024*	
C6	0.44843 (10)	0.56903 (15)	0.63656 (7)	0.0194 (2)	
H6A	0.4001	0.6329	0.6644	0.023*	
H6B	0.4825	0.4734	0.6721	0.023*	
C7	0.36711 (10)	0.50483 (14)	0.55465 (7)	0.0173 (2)	
H7	0.3549	0.5928	0.5116	0.021*	
C8	0.24722 (10)	0.45733 (15)	0.56663 (7)	0.0186 (2)	
H8A	0.2625	0.3806	0.6140	0.022*	
H8B	0.2107	0.5583	0.5813	0.022*	
C9	0.15852 (10)	0.37740 (14)	0.49344 (7)	0.0162 (2)	
C10	0.08491 (10)	0.47543 (14)	0.43015 (7)	0.0170 (2)	
C11	−0.00046 (10)	0.40041 (14)	0.36511 (7)	0.0177 (2)	
H11	−0.0496	0.4676	0.3226	0.021*	
C12	−0.01591 (10)	0.22964 (14)	0.36054 (7)	0.0175 (2)	
C13	0.05775 (10)	0.13441 (14)	0.42312 (7)	0.0178 (2)	
H13	0.0488	0.0177	0.4209	0.021*	
C14	0.14458 (10)	0.20477 (14)	0.48924 (7)	0.0172 (2)	
C15	0.21904 (11)	0.09380 (15)	0.55627 (7)	0.0229 (3)	
H15A	0.2038	−0.0219	0.5394	0.034*	
H15B	0.1984	0.1125	0.6072	0.034*	
H15C	0.3034	0.1187	0.5657	0.034*	
C16	−0.11163 (11)	0.15251 (15)	0.29073 (7)	0.0245 (3)	
H16A	−0.0934	0.0354	0.2861	0.037*	
H16B	−0.1153	0.2092	0.2391	0.037*	
H16C	−0.1884	0.1627	0.3015	0.037*	
C17	0.09396 (11)	0.66162 (14)	0.43230 (8)	0.0213 (3)	
H17A	0.0326	0.7081	0.3851	0.032*	
H17B	0.1726	0.6948	0.4297	0.032*	
H17C	0.0825	0.7028	0.4835	0.032*	

### Atomic displacement parameters (Å^2^)

**Table:**

	*U*^11^	*U*^22^	*U*^33^	*U*^12^	*U*^13^	*U*^23^
O1	0.0219 (4)	0.0226 (4)	0.0206 (4)	0.0023 (3)	0.0080 (4)	−0.0009 (3)
N1	0.0141 (4)	0.0170 (5)	0.0163 (5)	−0.0008 (4)	0.0017 (4)	0.0007 (4)
C1	0.0176 (5)	0.0214 (6)	0.0169 (5)	−0.0004 (4)	0.0010 (4)	0.0011 (4)
C2	0.0172 (6)	0.0247 (6)	0.0214 (6)	−0.0025 (5)	0.0022 (4)	−0.0019 (5)
C3	0.0249 (6)	0.0212 (6)	0.0227 (6)	−0.0065 (5)	0.0074 (5)	−0.0038 (5)
C4	0.0275 (6)	0.0180 (5)	0.0216 (6)	−0.0005 (5)	0.0066 (5)	0.0007 (5)
C5	0.0189 (6)	0.0187 (5)	0.0194 (6)	0.0029 (4)	0.0028 (4)	0.0000 (4)
C6	0.0185 (6)	0.0233 (6)	0.0164 (5)	−0.0042 (4)	0.0050 (4)	−0.0009 (4)
C7	0.0161 (5)	0.0185 (5)	0.0169 (5)	−0.0018 (4)	0.0045 (4)	−0.0011 (4)
C8	0.0175 (5)	0.0210 (5)	0.0179 (5)	−0.0027 (4)	0.0064 (4)	−0.0035 (4)
C9	0.0142 (5)	0.0177 (5)	0.0180 (5)	−0.0015 (4)	0.0067 (4)	−0.0027 (4)
C10	0.0167 (5)	0.0156 (5)	0.0205 (6)	−0.0002 (4)	0.0083 (4)	−0.0012 (4)
C11	0.0157 (5)	0.0185 (5)	0.0186 (5)	0.0011 (4)	0.0048 (4)	0.0012 (4)
C12	0.0158 (5)	0.0193 (5)	0.0181 (5)	−0.0018 (4)	0.0059 (4)	−0.0024 (4)
C13	0.0192 (6)	0.0140 (5)	0.0214 (6)	−0.0017 (4)	0.0079 (5)	−0.0014 (4)
C14	0.0164 (5)	0.0174 (5)	0.0187 (5)	0.0009 (4)	0.0068 (4)	0.0015 (4)
C15	0.0227 (6)	0.0204 (6)	0.0236 (6)	0.0005 (5)	0.0040 (5)	0.0033 (5)
C16	0.0256 (6)	0.0236 (6)	0.0209 (6)	−0.0052 (5)	0.0017 (5)	−0.0025 (5)
C17	0.0226 (6)	0.0152 (5)	0.0261 (6)	−0.0010 (4)	0.0072 (5)	−0.0009 (4)

### Geometric parameters (Å, °)

**Table:**

O1—C7	1.4233 (14)	C8—C9	1.5106 (15)
O1—H1	0.852 (9)	C8—H8A	0.9900
N1—C6	1.4687 (14)	C8—H8B	0.9900
N1—C1	1.4720 (14)	C9—C14	1.4062 (15)
N1—C5	1.4749 (14)	C9—C10	1.4079 (16)
C1—C2	1.5205 (16)	C10—C11	1.3923 (15)
C1—H1A	0.9900	C10—C17	1.5104 (15)
C1—H1B	0.9900	C11—C12	1.3933 (16)
C2—C3	1.5258 (17)	C11—H11	0.9500
C2—H2A	0.9900	C12—C13	1.3883 (16)
C2—H2B	0.9900	C12—C16	1.5068 (15)
C3—C4	1.5262 (17)	C13—C14	1.3955 (16)
C3—H3A	0.9900	C13—H13	0.9500
C3—H3B	0.9900	C14—C15	1.5092 (16)
C4—C5	1.5203 (17)	C15—H15A	0.9800
C4—H4A	0.9900	C15—H15B	0.9800
C4—H4B	0.9900	C15—H15C	0.9800
C5—H5A	0.9900	C16—H16A	0.9800
C5—H5B	0.9900	C16—H16B	0.9800
C6—C7	1.5265 (15)	C16—H16C	0.9800
C6—H6A	0.9900	C17—H17A	0.9800
C6—H6B	0.9900	C17—H17B	0.9800
C7—C8	1.5375 (15)	C17—H17C	0.9800
C7—H7	1.0000		
			
C7—O1—H1	108.5 (12)	C6—C7—H7	109.8
C6—N1—C1	109.65 (9)	C8—C7—H7	109.8
C6—N1—C5	109.73 (9)	C9—C8—C7	115.77 (9)
C1—N1—C5	109.36 (9)	C9—C8—H8A	108.3
N1—C1—C2	111.20 (9)	C7—C8—H8A	108.3
N1—C1—H1A	109.4	C9—C8—H8B	108.3
C2—C1—H1A	109.4	C7—C8—H8B	108.3
N1—C1—H1B	109.4	H8A—C8—H8B	107.4
C2—C1—H1B	109.4	C14—C9—C10	119.03 (10)
H1A—C1—H1B	108.0	C14—C9—C8	120.59 (10)
C1—C2—C3	111.12 (10)	C10—C9—C8	120.32 (10)
C1—C2—H2A	109.4	C11—C10—C9	119.67 (10)
C3—C2—H2A	109.4	C11—C10—C17	118.91 (10)
C1—C2—H2B	109.4	C9—C10—C17	121.40 (10)
C3—C2—H2B	109.4	C10—C11—C12	121.89 (11)
H2A—C2—H2B	108.0	C10—C11—H11	119.1
C2—C3—C4	110.11 (10)	C12—C11—H11	119.1
C2—C3—H3A	109.6	C13—C12—C11	117.82 (10)
C4—C3—H3A	109.6	C13—C12—C16	121.53 (10)
C2—C3—H3B	109.6	C11—C12—C16	120.64 (10)
C4—C3—H3B	109.6	C12—C13—C14	122.05 (10)
H3A—C3—H3B	108.2	C12—C13—H13	119.0
C5—C4—C3	110.84 (10)	C14—C13—H13	119.0
C5—C4—H4A	109.5	C13—C14—C9	119.52 (10)
C3—C4—H4A	109.5	C13—C14—C15	119.10 (10)
C5—C4—H4B	109.5	C9—C14—C15	121.35 (10)
C3—C4—H4B	109.5	C14—C15—H15A	109.5
H4A—C4—H4B	108.1	C14—C15—H15B	109.5
N1—C5—C4	111.78 (9)	H15A—C15—H15B	109.5
N1—C5—H5A	109.3	C14—C15—H15C	109.5
C4—C5—H5A	109.3	H15A—C15—H15C	109.5
N1—C5—H5B	109.3	H15B—C15—H15C	109.5
C4—C5—H5B	109.3	C12—C16—H16A	109.5
H5A—C5—H5B	107.9	C12—C16—H16B	109.5
N1—C6—C7	114.35 (9)	H16A—C16—H16B	109.5
N1—C6—H6A	108.7	C12—C16—H16C	109.5
C7—C6—H6A	108.7	H16A—C16—H16C	109.5
N1—C6—H6B	108.7	H16B—C16—H16C	109.5
C7—C6—H6B	108.7	C10—C17—H17A	109.5
H6A—C6—H6B	107.6	C10—C17—H17B	109.5
O1—C7—C6	107.70 (9)	H17A—C17—H17B	109.5
O1—C7—C8	111.31 (9)	C10—C17—H17C	109.5
C6—C7—C8	108.36 (9)	H17A—C17—H17C	109.5
O1—C7—H7	109.8	H17B—C17—H17C	109.5
			
C6—N1—C1—C2	179.35 (9)	C14—C9—C10—C11	0.41 (16)
C5—N1—C1—C2	−60.29 (12)	C8—C9—C10—C11	−176.83 (10)
N1—C1—C2—C3	57.57 (13)	C14—C9—C10—C17	178.63 (10)
C1—C2—C3—C4	−52.80 (13)	C8—C9—C10—C17	1.39 (16)
C2—C3—C4—C5	52.23 (13)	C9—C10—C11—C12	0.14 (17)
C6—N1—C5—C4	−179.48 (9)	C17—C10—C11—C12	−178.12 (11)
C1—N1—C5—C4	60.20 (12)	C10—C11—C12—C13	−0.52 (17)
C3—C4—C5—N1	−56.81 (13)	C10—C11—C12—C16	178.08 (10)
C1—N1—C6—C7	−155.49 (10)	C11—C12—C13—C14	0.34 (16)
C5—N1—C6—C7	84.37 (12)	C16—C12—C13—C14	−178.24 (11)
N1—C6—C7—O1	81.03 (12)	C12—C13—C14—C9	0.20 (17)
N1—C6—C7—C8	−158.46 (9)	C12—C13—C14—C15	178.26 (10)
O1—C7—C8—C9	−56.80 (13)	C10—C9—C14—C13	−0.57 (16)
C6—C7—C8—C9	−175.04 (10)	C8—C9—C14—C13	176.66 (10)
C7—C8—C9—C14	98.81 (13)	C10—C9—C14—C15	−178.59 (10)
C7—C8—C9—C10	−84.00 (13)	C8—C9—C14—C15	−1.36 (16)

### Hydrogen-bond geometry (Å, °)

**Table:**

*D*—H···*A*	*D*—H	H···*A*	*D*···*A*	*D*—H···*A*
O1—H1···N1^i^	0.85 (1)	2.07 (1)	2.880 (1)	158.(2)

Symmetry codes: (i) −*x*+1, −*y*+1, −*z*+1.
